# Designing strategies and enhancing mechanism for multicomponent high-entropy catalysts

**DOI:** 10.1039/d2sc06403k

**Published:** 2023-01-03

**Authors:** Haitao Xu, Zeyu Jin, Yinghe Zhang, Xi Lin, Guoqiang Xie, Xingjun Liu, Hua-Jun Qiu

**Affiliations:** a School of Materials Science and Engineering, Dongguan University of Technology Dongguan 523808 China; b School of Materials Science and Engineering, Harbin Institute of Technology (Shenzhen) Shenzhen 518055 China qiuhuajun@hit.edu.cn; c School of Science, Harbin Institute of Technology (Shenzhen) Shenzhen 518055 China

## Abstract

High-entropy materials (HEMs) are new-fashioned functional materials in the field of catalysis owing to their large designing space, tunable electronic structure, interesting “cocktail effect”, and entropy stabilization effect. Many effective strategies have been developed to design advanced catalysts for various important reactions. Herein, we firstly review effective strategies developed so far for optimizing HEM-based catalysts and the underlying mechanism revealed by both theoretical simulations and experimental aspects. In light of this overview, we subsequently present some perspectives about the development of HEM-based catalysts and provide some serviceable guidelines and/or inspiration for further studying multicomponent catalysts.

## Abstract 2

In this review, HEM-based catalyst designing strategies were summarized, including nanostructure design, defect engineering, strain effect, composition regulation, and theoretical calculation/prediction.

## Graphical abstract

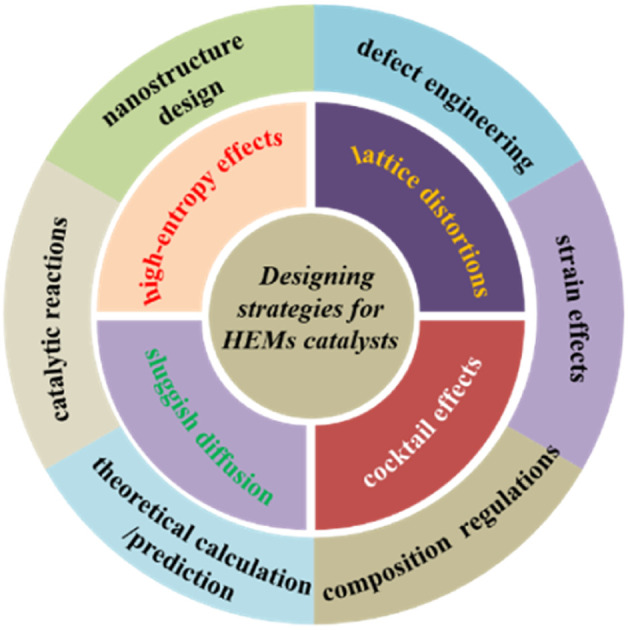

## 1. Introduction

In 2004, the high-entropy concept from equiatomic multicomponent alloys was put forward in two pioneering research studies, in which a CuCoNiCrAlFe alloy with an equal atomic ratio was prepared by arc melting the constituent elements with high configuration entropy.^1^ After that, multi-principal element alloys with a solid solution structure were classified as high-entropy alloys (HEAs).^2,3^ Generally, different from traditional alloys, HEAs could conquer the tremendous immiscible gap between various metallic elements to form single phase solid solutions rather than heterogeneous systems.^4–6^ Based on the entropy stabilization effect, sluggish diffusion, tailored compositions, and cocktail effects, a series of interesting properties were observed such as enhanced hardness and strength, as well as resistance to wear, oxidation, corrosion, *etc.*^7–11^ Besides getting great attention as structural materials, HEAs have been tried in catalysis. Nanostructured noble metals and their alloys are the most prevalent and effective materials for catalysis. Therefore, noble-metal-based HEAs were first investigated and received extensive attention worldwide. In recent years, a series of high-entropy materials (HEMs), including alloys,^12^ oxides,^13^ layered double hydroxides,^14^ perovskite oxides,^15^ zeolites,^16^ phosphates,^17^ sulfides,^18^ metal–organic frameworks (MOFs),^19–21^ and (oxy)hydroxides,^22^ have been prepared, which show promising applications in various areas, such as heat resistance,^23^ hydrogen storage,^24^ batteries,^25,26^ photothermal conversion,^27^ catalysis/electrocatalysis,^28^*etc.* From the catalytic application point of view, the random distribution of different metal elements in HEMs could contribute to transcendental homogeneity, leading to an unexpected synergistic effect to stimulate the kinetic barrier for adsorption/desorption, further leading to promising properties.^29–32^ Besides, their adjustable compositions, electronic structure, and good durability in corrosive electrolytes make HEMs promising candidates for advanced catalysis/electrocatalysis.

**Fig. 1:**
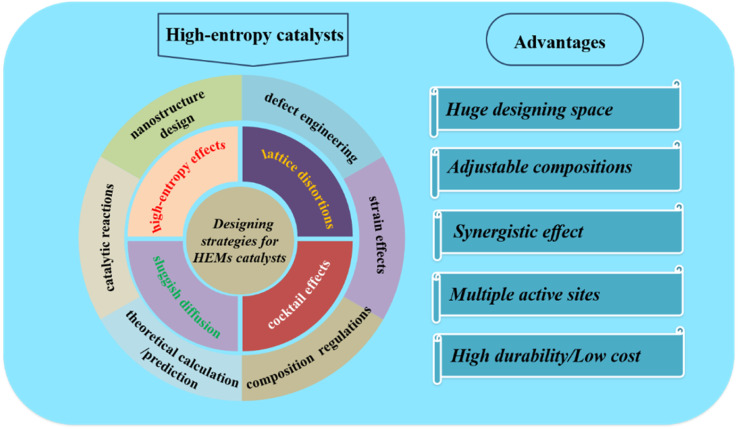
Overview of the properties of high-entropy materials and their designing strategies.

Recently, several papers have reviewed HEMs, their preparation methods and applications in different areas.^33–38^ However, the designing and regulating strategies for improving the catalytic performances of HEMs have been rarely involved. Meanwhile, an elaborate comparison of different designing strategies for high-entropy-based catalysts is urgently needed due to the rapid development in this area, so as to discover synergistic effects by combining different elements for different reactions. In this perspective, as illustrated in Fig. 1, we will review the reported many designing and regulating strategies for HEM-based catalysts. Then, based on these achievements, we will discuss some viewpoints about the further development on multicomponent high-entropy catalysts. We hope that the insights and critical suggestions presented here could inspire researchers to develop more advanced high-entropy catalysts.

## 2. Nanostructure design

As is well known, nanostructure design is a very effective route to prepare high-performance catalysts considering the high specific surface area, abundant low-coordinated sites, surface defects and surface strain effects.^39–43^ Moreover, the nanoscale size effect, structure effect, crystal facet effect, *etc.*, make nanomaterials particularly interesting for catalysis. Hence, purposefully designing a nanostructure is one of the most effective pathways to regulate the overall catalytic performance of HEMs.

### 2.1. Nanoparticle design

**Fig. 2:**
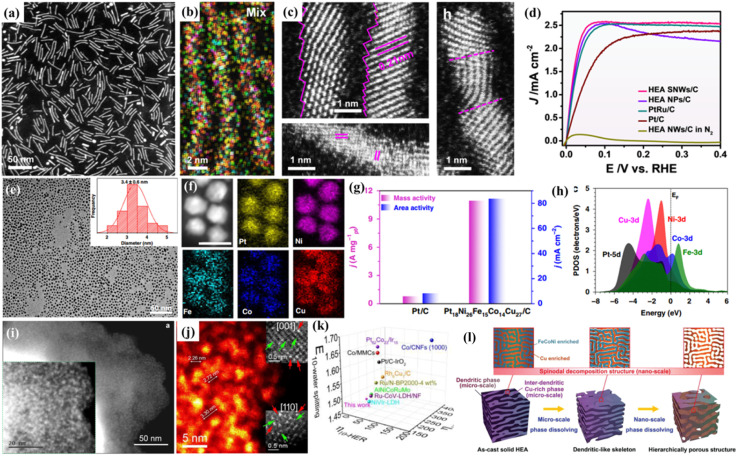
(a) HAADF-STEM image; (b) HAADF-STEM-EDS elemental mappings; (c) atomic scale STEM images of PtRuNiCoFeMo HEA nanowires; (d) polarization curves in a H_2_-saturated KOH electrolyte; (a–d) adapted with permission from ref. 48. Copyright 2021, Springer Nature. (e) TEM image of Pt_18_Ni_26_Fe_15_Co_14_Cu_27_ nanoparticles; (f) the corresponding elemental mapping of Pt_18_Ni_26_Fe_15_Co_14_Cu_27_ nanoparticles; (g) comparison of area activity and mass activity values for the HER at −70 mV *vs.* RHE; (h) the PDOSs of the HEA; (e–h) adapted with permission from ref. 49 copyright 2020, Springer Nature. HAADF-STEM image (i) and high-magnification HADDF-STEM image (j) of the 12 element HEAs; (k) comparisons of the overpotentials at 10 mA cm^−2^ for the HER, OER, and overall water splitting with literature data; adapted with permission from ref. 59, copyright 2022, American Chemical Society. (l) The schematic diagram for the formation of the hierarchical porous structure *via* dealloying the FeCoNiCu HEA with microscale phase separation and nanoscale spinodal decomposition; adapted with permission from ref. 60, copyright 2022, Wiley-VCH.

It has been widely acknowledged that precise control of the structure of HEMs at the nanoscale is an effective approach to moderate their catalytic performances, which is due to the fact that the properties of HEMs are influenced largely by their crystal structures, such as crystal sizes, exposed crystal faces, and surface strains.^44–47^ Recently, Huang's group synthesized distinctive PtRuNiCoFeMo HEA nanowires for an alkaline hydrogen oxidation reaction (HOR) (Fig. 2a and b).^48^ With an ultrafine diameter of 1.8 ± 0.3 nm, the HEA nanowires have distorted surface atomic structures with a rich atomic step and defect-rich lattice mismatch (Fig. 2c). Moreover, the electron transfer from Ni, Co, Fe and Mo to Ru in the HEA nanowires can moderate the binding strengths of reactants/intermediates during the HOR. Thanks to the sub-nanometer feature and electron transfer effect, the mass activity and specific activity of the HEA nanowires reach 6.75 A mg_Pt+Ru_^−1^ and 8.96 mA cm^−2^ for the HOR, which are 4.1/2.4 and 19.8/18.7 times higher than those of commercial PtRu/C and Pt/C, respectively (Fig. 2d).

Similarly, Wang and coworkers reported the synthesis of uniform and ultrasmall (∼3.4 nm) HEA nanoparticles (Pt_18_Ni_26_Fe_15_Co_14_Cu_27_) by a simple low-temperature oil phase strategy (Fig. 2e and f).^49^ The catalyst shows an ultrasmall overpotential of 11 mV at 10 mA cm^−2^ for the HER and 15.04 A mg_Pt_^−1^ is achieved for the methanol oxidation reaction (MOR) in alkaline media (Fig. 2g). DFT calculations show multi-active sites for both proton and intermediate transformation during both the HER and the MOR. Meanwhile, the nanoscale HEA surface provides fast site-to-site electron transfer for both the reduction and oxidation reactions (Fig. 2h). By *in situ* growth of quinary metal–organic frameworks (MOFs) on carbon cloth, followed by pyrolysis reduction, Wen and coworkers prepared a HEA-CoNiCuMnMo electrode, which exhibits a high performance for the glycerol oxidation reaction (GOR) with a low overpotential and high selectivity toward formate production.^50^ Machine learning-based Monte Carlo simulation was used to study the surface atomic configurations of CoNiCuMnMo, which reveals Mo sites coordinated by Mn, Mo, and Ni as the catalytically active sites.

Considering the high performance of Pt-group noble metals, Wu *et al.*^46^ prepared HEA nanoparticles containing all six Pt-group metals (PGM-HEA), which have different adsorption sites on their surfaces and may be ideal candidates for complex reactions. PGM-HEA exhibits greatly enhanced catalytic activity for the ethanol oxidation reaction (EOR) with a 12-electron/12-proton transfer process. The performance is 2.5 (3.2) and 12.8 (3.4) times higher than that of Pd/C and Pt/C catalysts in terms of surface (mass) activity, respectively. By both X-ray photoelectron spectroscopy and DFT calculations, they later revealed that some same-element atoms in HEAs have different local density of states (LDOS) profiles, whereas some different element atoms have similar LDOS profiles. The results indicate that the electronic structures of atoms in HEAs are highly adjustable and they can lose their original identity.^51^

Xia and coworkers synthesized ultrasmall sub-2 nm HEA nanoparticles (NiCoFePtRh), which achieve an ultrahigh mass activity of 28.3 A mg^−1^ at −0.05 V for the HER in 0.5 M H_2_SO_4_ solution, which is 40.4 times larger than that of the commercial Pt/C catalyst.^52^ Operando X-ray absorption spectroscopy and theoretical calculations reveal that Rh and Pt are the main and direct active sites. Fe/Co/Ni can effectively adjust the electronic structures of Pt/Rh atoms and increase the entropy of the system.

Rational and controllable synthesis of multicomponent high-entropy materials with specific nanostructures has been an interesting and hot topic in catalysis. Quite recently, Guo and coworkers reported a general strategy to prepare ultrathin noble metal-based HEA nano-ribbons *via* a galvanic exchange reaction between different metal precursors and an Ag nanowire template.^53^ This synthetic method also enables flexible control on the elements and concentrations of the HEAs. The PtPdAgRuIr combination exhibits a much higher ORR performance when compared with the Pt/C catalyst. DFT calculations reveal that the high electrochemical performance is due to the strong reduction capability of the high-concentration reductive elements in the HEA, while the other elements guarantee the site-to-site efficient electron transfer. 2D ultrathin HEAs have also been prepared by simple chemical reduction methods.^54^ Combined with computer-facilitated screening, the developed multicomponent PdMoGaInNi HEA nanosheets exhibit a high HER activity with a low overpotential of 13 mV at 10 mA cm^−2^, even better than commercial Pd/C and Pt/C.^54^

Non-noble metal-based alloy nanoparticles have also been used as a core to decorate noble metal catalysts to enhance the stability and reduce the cost. For example, Pd can be directly decorated on a non-noble HEA (FeCoNiSn) nanoparticle surface through the galvanic exchange reaction. Core–shell-structured nanoparticles are highly active for the EOR owing to the unique high-entropy coordination environment for surface Pd.^11^

### 2.2. Nanoporous/hollow structure design

Nanoporous/hollow materials have gained extensive attention in the field of catalysis due to their large specific surface area, high surface-to-volume ratio, enhanced mass transfer, *etc.* For example, nanoporous metals obtained by dealloying have been widely studied as advanced catalysts.^55,56^ Moreover, the top-down dealloying strategy also has advantages in terms of large-scale preparation, high repeatability and no need for any organic chemicals. Thus, we recently prepared a series of nanoporous HEAs or HEOs with different pore structures by dealloying.^57–59^ Al-based precursor alloys are widely used in our work due to their low cost, easy and selective dissolution in alkaline solutions, and other metal elements still remain during the corrosion process. In our designed Al-based precursor alloys, they contain uniformly mixed two phases (pure Al phase and Al_3_X phase), and the dealloying would result in a hierarchical nanoporous structure with both big pores from the removal of the pure Al phase and small nanopores from the dealloying of the Al_3_X intermetallic phase. Recently, to fabricate free-standing nanoporous HEAs, single phase Mn-based precursor alloys are designed.^59^ The Mn-based precursor alloy system is capable of incorporating different metal elements into one FCC phase and as a result, free-standing nanoporous HEAs composed of up to twelve or even sixteen metallic elements are obtained.^59^ Thanks to the multi-element surface, the freestanding 12-component nanoporous HEA exhibits multiple catalytic activities such as OER, HER, and ORR activities (Fig. 2i and j). The high electrochemical catalytic performance indicates the possible synergistic effects by combining different elementals in one system (Fig. 2k). Similarly, Lu's group reported a free-standing dealloyed nanoporous HEA electrocatalyst for high-efficiency hydrogen production (Fig. 2l).^60^ They found that in the quaternary FeCoNiCu precursor alloy, the miscibility gap between Cu and other metallic elements contributes to microscopic phase separation and nanoscale spinodal decomposition. As a result, a hierarchical nanoporous system was obtained and displayed excellent HER performances, with an extremely low overpotential of 42.2 mV (10 mA cm^−2^) and a small Tafel slope of 31.7 mV dec^−1^.

Combining multicomponent metal elements in one hollow nanostructure is promising for the production of highly efficient and cost-effective catalysts. *Via* a facile one-pot solvothermal method, Zuo *et al.*^61^ synthesized PdCuMoNiCo HEA nanoparticles with a nano-hollow spherical structure, which results in a much higher specific surface area compared with their solid counterparts. When used as a bi-functional electrocatalyst for both formic acid oxidation (FAO) and the oxygen reduction reaction (ORR), Pd is the active center and the other elements act as co-catalysts to adjust the adsorption energy of the intermediates and accelerate the catalyzed reaction.^61^ In another study, Hu and coworkers developed a continuous “droplet-to-particle” strategy for the formation of hollow HEA nanoparticles, which is enabled *via* the decomposition of a gas-blowing agent of citric acid.^62^ The generated Co_*x*_/H_2_O gas would “puff” the droplet during heating, which is followed by the fast reduction of the mixed metal precursors and growth of multicomponent HEA particles. When used as a Li–O_2_ battery catalyst, the prepared RuIrFeCoNi hollow nanoparticles generate a record-high current density of 2000 mA g_cat._^−1^ and show high cycling stability.^62^

### 2.3. High-entropy-based composite design

#### 2.3.1 HEM/metal composite

The entropy-driven structural stability and rich coordination environment in HEMs not only endow them with high catalytic activity, but also make them suitable to act as co-catalyst or catalyst supports in many reactions, such as photocatalysis, thermal-catalysis and electrocatalysis. For example, due to the high structural entropy, high-entropy oxides (HEOs) can be an excellent support to disperse and stabilize noble-metal nanoclusters or even single atoms.^63–65^ High-entropy supports with a tunable element combination and electronic structure can also be used to modulate the electronic structure of noble metal-based active sites and *vice versa*.^66–68^

**Fig. 3:**
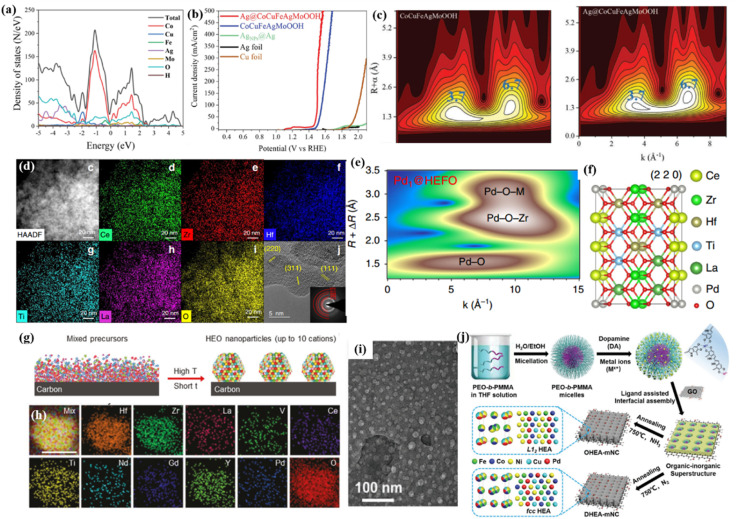
(a) TDOS and PDOS plots for Ag@CoCuFeAgMoOOH; (b) LSV curves of the prepared samples; (c) wavelet transform analysis of CoCuFeAgMoOOH and Ag@CoCuFeAgMoOOH, respectively; (a–c) adapted with permission from ref. 67. Copyright 2022, Wiley-VCH. (d) EDS mapping of HEFO; (e) the wavelet transforms from experimental data for Pd@HEFO; (f) schematic model of Pd@HEFO (220). (d–f) Adapted with permission from ref. 66, copyright 2020, Springer Nature. (g) The synthesis of carbon-supported HEO nanoparticles using our rapid high-temperature method; (h) elemental mapping of 10-HEO/C after the chronoamperometry test. (g and h) Adapted with permission from ref. 75, copyright 2021, Wiley-VCH. (i) TEM image of the as-synthesized organic–inorganic superstructure; (j) schematic illustration for the construction of structurally ordered HEA NPs on 2D nitrogen-rich mesoporous carbon nanosheets; (i and j) adapted with permission from ref. 76, copyright 2022, Wiley-VCH.

Specifically, noble metal single atoms/nanoparticles could lead to an unexpected synergistic effect to stimulate the kinetic barrier for adsorption/desorption, thereby affecting the intrinsic activity.^68^ Recently, Zhang *et al.*^67^ introduced Ag clusters as an electron donor to activate the metal sites in high-entropy CuCoFeAgMo (oxy)hydroxides for an efficient OER process. They proposed that metal–oxygen hybridization for each metal site could act as an effective descriptor to illustrate the specific catalytic activity of different metal sites. Meanwhile, they demonstrated that the Ag site with a lower limiting energy barrier in CuCoFeAgMo (oxy)hydroxides could boost the OER activity of quaternary CuCoFeAgMo (oxy)hydroxides (Fig. 3c). Furthermore, the Ag clusters with higher Fermi levels were verified to act as electron donors to increase the hybridization between the O 2p states of the intermediates and the d states of Co, Cu, Fe, and Mo (Fig. 3a and c). Unsurprisingly, Ag@CoCuFeAgMoOOH displayed outstanding and steady OER performances, such as a low overpotential of 270 mV at a current density of 100 mA cm^−2^, a Tafel slope of 35.3 mV dec^−1^, and excellent stability over 50 h (Fig. 3b).

Recently, a mechanochemical method was introduced to fabricate Pd single atoms substituted on a high-entropy fluorite oxide (Pd@HEFO) composite by simple mechanical milling (Fig. 3d).^66^ About 6.44% surface Pd atoms verified by CO chemisorption inserted into both the surface and the bulk HEFO phase. In contrast with CeO_2_, HEFO afforded the elevated reducibility of lattice oxygen. More importantly, the bonding effect of stable Pd–O–M endows Pd@HEFO with high low temperature CO oxidation activities, as well as remarkable thermal stability (Fig. 3e and f). The same mechanochemical method was also used to synthesize Pt or Ru single atoms/nanoparticles@(NiMgCuZnCo)O_*x*_ for the hydrogenation of atmospheric CO_2_ to CO.^69^

By precursor alloy design and the top-down dealloying strategy, we also prepared a nanoporous HEO decorated with Pt nanoparticle (Pt@HEO) composite.^70^ We found that the incorporation of Pt in the Al_3_CoFeMoCr intermetallic phase is the key for the formation of uniformly and inherently doped Pt clusters (∼2 nm) on nanoporous (AlCoFeMoCr)_3_O_4−*x*_. For the OER/ORR bifunctional electrocatalyst design, we found that the incorporation of Pt clusters can clearly enhance the OER activity of the HEO support although Pt is mainly for the ORR and the HEO support can also enhance the ORR activity of Pt.^70^ The experimentally observed synergistic effect between Pt and the HEO is also confirmed by DFT calculation. Moreover, the element compositions of both the HEO support and the noble metal nanocluster can be easily tuned, which will be used for developing more advanced and multifunctional catalysts with high durability considering that both the HE-support and the nanoclusters are catalytically active.^71^

#### 2.3.2 HEM/carbon composite

Multicomponent high-entropy materials could be thermodynamically stable or metastable in the preparation process. In the latter case, one needs to fabricate HEMs under conditions such as high temperature or high chemical potential, which is conducive to the formation of a single-phase solid solution phase. Hence, the same effective methods have been developed to address the poor stability of HEMs. Recently, a rapid high-temperature synthesis strategy is developed by Hu's group to quench and maintain the single phase of HEMs by kinetic trapping. On the other hand, the fabrication of carbon-supported composites is one of the most common strategies for advanced electrocatalyst design.^72,73^ It could not only improve the electrical conductivity of the parent material, but also provides stable chemical and electrochemical reaction interfaces.^74^ For example, Hu's group adopted a carbon shock method to fabricate HEA and HEO nanoparticles monodispersed on the carbon substrate (Fig. 3g).^75^ The high synthesis temperature not only guarantees uniform alloy structures without phase separation, but also reinforces the bonding effects between the HEA/HEO nanoparticles and carbon substrate to further enhance the structural durability (Fig. 3h). Specifically, the as-prepared 10-component HEOs/C delivers a comparable ORR activity to Pd/C with a much less Pd loading amount and sharply increased durability (86% retention after 100 h).

Zhu *et al.*^76^ prepared structurally ordered HEA intermetallic NPs on thin N-doped mesoporous carbon by combining a ligand-assisted interfacial assembly with NH_3_ annealing (Fig. 3i and j). The micelle template ensures the formation of uniform HEA nanoparticles with sizes of ∼20 nm. Thanks to the structural design, the PdNiCoFeCu HEA/C composite exhibits a better ORR performance than commercial Pt/C in alkaline solutions. Besides the single attaching HEM on carbon supports, the further coating of high-entropy catalysts with a thin carbon film should further enhance the overall catalytic durability.

## 3. Defect engineering

The physical and chemical properties of HEMs directly affect the surface adsorption and desorption of intermediates during a catalytic process.^77–79^ Thus, surface defect engineering is a highly significant and useful strategy to enhance the catalytic activity by regulating the surface electronic structures of HEMs. Although atomically mixing multiple elements makes it easy for the existence of abundant defects, the strategies to introduce defects is disparate with regard to various high-entropy compounds, such as hydroxides, perovskite oxides, layered double hydroxides (LDHs), and MOFs. In this section, we summarized the synthetic strategies for creating defects in HEMs and discussed the defect structure–activity relationship.

**Fig. 4:**
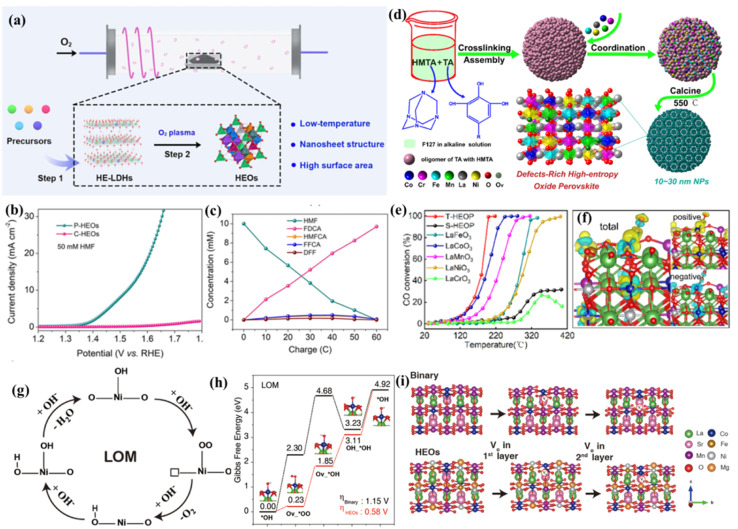
(a) The low-temperature plasma strategy for HEOs; (b) LSV curves of P-HEOs and C-HEOs; (c) concentration changes of HMF and its oxidation products during HMF electrooxidation; (a–c) adapted with permission from ref. 82. Copyright 2021, Wiley-VCH. (d) Illustration of the synthesis of HEOP NPs *via* a metal–phenolic coordination assembly strategy; (e) light-off curves for CO conversions on T-HEOP, S-HEOP, and single-oxide perovskites; (f) charge density difference plots of T-HEOP with CO molecules adsorbed on Co atoms. (d–f) Adapted with permission from ref. 83, copyright 2022, American Chemical Society. (g) The schematic of the LOM pathway; (h) LOM on HEOs and a binary surface at a potential of 0 V, and the absorption model on HEOs in the inset, respectively; (i) the model of oxygen vacancy formation on the first layer and second layer slab of binary and HEO surfaces. (g–i) Adapted with permission from ref. 89, copyright 2022, Wiley-VCH.

Ar plasma etching has been widely adopted to introduce defects in metal oxides and hydroxides.^80,81^ Recently, Wang's group expands the application range of this technique to high-entropy LDHs. For example, to create abundant active surface sites, oxygen vacancies were introduced into HEO nanosheets by a low temperature plasma strategy.^14^ The inelastic collision of high-energy electrons and oxygen molecules will transfer energy to oxygen molecules, contributing to the formation of provocative oxygen species with much higher chemical activity in comparison with initial oxygen. To be specific, high-entropy LDHs acting as a precursor could be transformed into HEOs with the help of low-temperature plasma techniques, endowing the as-prepared HEO nanosheets with vast oxygen vacancies and a high surface area (Fig. 4a). As a proof of concept, the prepared HEO nanosheets were adopted for 5-hydroxymethylfurfural (HMF) electrooxidation and displayed efficient catalytic performances with small overpotentials (Fig. 4b and c).^82^

Recently, a novel metal–tannin coordination assembly tactic is developed to fabricate high-entropy perovskite oxide nanoparticles with ample oxygen vacancies. In this work, Chen *et al.*^83^ indicated that tannin acid can crosslink with methenamine in basic solution, and tannin acting as the building block assembles with F127 through a hydrogen bond, resulting in low-polymerized structures (Fig. 4d). After that, various metal cations will coordinate with OH^−^ of the above oligomers by metal ligand chelation. Then, the precursor is transformed into high-entropy perovskite oxides by calcining in air. This specific metal–tannin coordination effect endows high-entropy perovskite oxide nanoparticles with a small particle size, abundant oxygen vacancies, high specific surface areas and enhanced catalytic performance for CO oxidation (Fig. 4e and f).

Recently, high-entropy MOFs (HE-MOFs) with incorporated multielement MOF nodes were fabricated.^19,21^ HE-MOFs possess more than five metal components, rich porosity, super-large specific surface areas, and abundant unsaturated metal nodes. Chen's group developed a solvent method to introduce cation defects into NiCoFeZnV-based HE-MOFs.^84^ They found that cationic vacancies generated by treatment in acidic aqueous solution would change the electronic structure of HE-MOFs, which finally accelerates the catalytic kinetic process for the nitrogen reduction reaction (NRR).

Lattice oxygen activation has been proven to be an effective strategy for electrocatalytic reactions, such as the OER.^85–88^ For example, scores of studies have verified that the lattice oxygen participation mechanism (LOM) delivers faster OER kinetics and lower energy barriers in comparison with the traditional adsorbate evolution mechanism (Fig. 4g). However, this strategy challenges the crystal structure stability of catalysts due to the reversible insertion/de-intercalation of lattice oxygen. Fortunately, the in-depth study of HEOs comes up with a new method for lattice oxygen activation. In a recent study, Sun's group developed a novel high-entropy perovskite cobaltate with five equimolar metals in the B-site *via* a simple sol–gel method.^89^ Comprehensive X-ray spectroscopic characterization manifests the reconstruction of the charge states and the change of the electronic configuration of oxygen sites, which jointly contributes to the improvement of intrinsic OER activities (Fig. 4h and i). As a result, a small overpotential of 320 mV at a current density of 10 mA cm^−2^ and a small Tafel slope of 45 mV dec^−1^ are achieved. The experimental results and theoretical calculations demonstrate that the high configuration entropy can facilitate the formation of oxygen vacancies, drastically skewing the OER mechanism from the adsorbate evolution mechanism to the more beneficial LOM pathway.

## 4. Strain effects

Surface strain is another important factor influencing the bond effect between the reaction intermediate and catalyst surface.^90,91^ Several research studies have been carried out to understand how surface strain influences the adsorption energy of intermediates, and the corresponding research findings have stimulated numerous original HEM design thoughts to moderate the dynamic process of HEM catalysts.^85–88^ For example, the incorporation of different atoms under a nanostructured noble metal surface (such as Pt) would clearly affect the surface strain and modify the catalytic activity. We found that the incorporation of Ni, Cu, and Mn atoms under a Pt surface would greatly enhance the ORR activity probably due to the surface strain effect.^92^ Since electrochemical dealloying in acidic solutions cannot completely remove the surface non-noble metals such as Mn, the ligand effect may also exist.

Wang and coworkers reported that PtFeCoNiCu HEA nanoparticles after 700 °C heat treatment (HEA-700) showed 0.94% compressive strain compared with that treated at 400 °C (HEA-400).^93^ HEA-700 exhibits a higher specific activity than HEA-400, which is due to a shorter Pt–Pt bond distance in HEA-700 resulting from the compressive strain. The non-noble Fe, Co, Ni, and Cu atoms in the core would cause compressive strain and down shift d-band centers *via* electron transfer to the surface Pt layer. Clausen *et al.*^94^ statistically analyzed the adsorption energy of *OH and *O on RuIrPtRhPd and PdPtAgAuCu HEAs with respect to the lattice constants of the alloys and the surface of each individual binding site. They found that the lattice distortion would mitigate the local strain effect on the adsorption energy as the atomic environment surrounding the binding atom(s) settles into a relaxed structure. As a result, the authors put forward that the local strain will negligibly influence the activity of the HEA surface, and the broadening of adsorption energy distribution is mainly owing to the neighboring atoms perturbing the electronic environment of the binding site. Therefore, the choice of constituent elements should be guided by the electronic perturbation of the ligand effect to obtain a target surface reactivity. It should be pointed out that the calculations usually use a uniform alloy structure, which is quite different from experimental conditions where core–shell structured nanoparticles with an almost pure noble metal shell are formed.

## 5. Composition regulations

One of the biggest advantages of multicomponent HEMs is that the element compositions/combinations are tunable, which provides a large design space for designing/screening effective catalysts for different reactions.^95–97^ Especially, the multicomponent induced synergistic effect and cocktail effect make multicomponent catalysts intriguing. Different from HEAs, multicomponent high-entropy compounds are made up of both anions and metal cations. Thus, in theory, both the anions and cations can be tuned to adjust the surface electronic structure and catalytic activities. So far, reported high-entropy compounds include oxides, layered double hydroxides, perovskite fluorides, carbides, nitrides, sulfides and (oxy)hydroxides.

### 5.1. Cation regulation

**Fig. 5:**
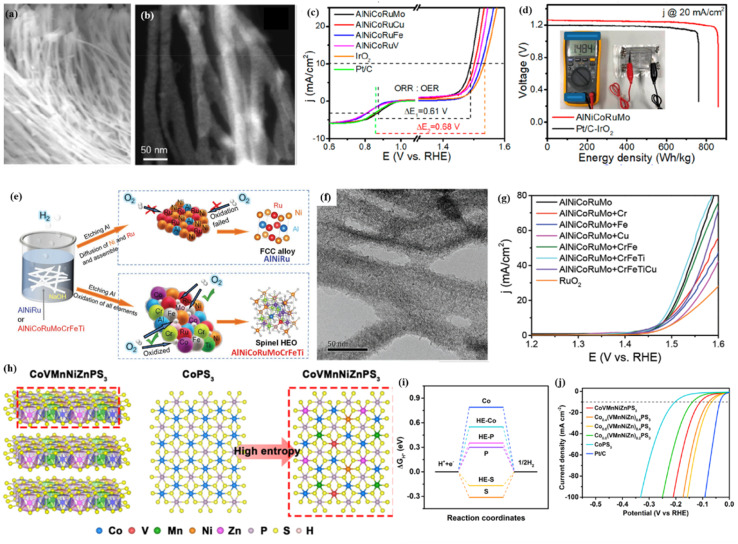
SEM (a) and HAADF-STEM (b) images of the dealloyed AlNiCoRuMo nanowires; (c) ORR/OER bifunctional LSV curves of different electrodes; (d) the discharge curves of the batteries catalyzed by AlNiCoRuMo and Pt/C–IrO_2_ under 20 mA cm^−2^; (a–d) adapted with permission from ref. 12. Copyright 2020, American Chemical Society. (e) Schematic explanation of the formation of FCC structured alloy and low-crystallized spinel oxides; (f) TEM image of the dealloyed eight-component HEO; (g) OER polarization curves of the as-prepared samples; (e–g) adapted with permission from ref. 98. Copyright 2022, Wiley-VCH. (h) Crystal structure of CoVMnNiZnPS_3_ with a monoclinic structure; and the structural polymorphs of CoPS_3_ and CoVMnNiZnPS_3_ along the top views, respectively; (i) HER free-energy diagram of the corresponding edge sites in CoVMnNiZnPS_3_; (j) LSV curves of the prepared samples; (h–j) adapted with permission from ref. 99. Copyright 2022, American Chemical Society.

Cation regulation has been widely employed to achieve the best catalytic performance for transition metal oxides, sulfides, and phosphides. For example, to achieve a high-performance electrocatalyst for the OER, we prepared a series of nanoporous alloys with surface spinel oxides by dealloying designed Al-based precursor alloys.^58^ By a detailed comparison study, we found that quinary AlNiCoFeCr and AlNiCoFeMo have similarly high OER performance, clearly better than the selected ternary AlNiFe, quaternary AlNiCoFe and many other quinary samples (Fig. 5a and b). It is known that noble metals are highly active for many reactions. For the sake of creating highly active and multifunctional catalysts, we then incorporated Ru into a nanoporous alloy/oxide system by the same dealloying method. Interestingly, we found that the quinary AlNiCoRuMo sample exhibited much better activities for the HER, OER and ORR when compared with other samples such as ternary AlNiRu, quaternary AlNiCoRu, and other quinary samples (Fig. 5c and d).^12^ It should also be mentioned that the quinary sample only contains 20 at% Ru. Thus, the high performance and low Ru content make the multicomponent high-entropy design very promising for developing advanced catalysts with a low cost. At that time, we have to ask one question. Is quinary AlNiCoRuMo the best combination and is it possible to further increase the catalytic performance by further increasing the component number? To answer this question, we then added more elements to the quinary AlNiCoRuMo combination.^98^ We found that by adding Fe, Cr, and Ti to quinary AlNiCoRuMo, forming an 8-component AlNiCoRuMoFeCrTi HEO, its OER and ORR activities are comparable with those of quinary AlNiCoRuMo (Fig. 5e and f). By a detailed comparison study, we found the underlying Cr–Fe synergistic effect on regulating the electronic structure of the active sites such as Co and Ru sites. The well-retained catalytic activities and much reduced Ru content (from 20 at% to 12.5% in theory) make the 8-component sample even more promising (Fig. 5g). We also found that when the number of metallic elements increased from five to eight, the optimized AlNiCoRuMoCrFeTi HEOs turn into amorphous-like oxides probably resulting from the increased entropy and limited surface diffusion. DFT calculation reveals that the interaction of different metal cations would tune the electronic structure of the active sites such Ni, Co and Ru, making them more active for catalytic reactions (OER/ORR).

In another study, Schuhmann and coworkers investigated multicomponent noble metal-free transition metal nanoparticle libraries and found that quinary CrMnFeCoNi exhibited surprisingly high ORR activity in alkaline solutions, which is comparable with pure Pt.^2^ Then, they carried out systematic removal of each component from the quinary alloy nanoparticle and found an obvious activity drop. The result shows the importance of synergistic mixing of five components in one system and the altered electronic properties by five-component interaction would overcome the limitation of single components. Thus, the multicomponent design strategy provides theoretically unlimited possibilities for developing advanced catalysts.

### 5.2. Anion regulation

Besides the widely used cation regulation strategy, anion regulation has been tried to prepare new types of high-entropy compounds, including phosphorus–boron,^99^ sulfide,^100^ and phosphorus–sulfide.^101^ Recently, as illustrated in Fig. 5h, Wang *et al.*^99^ reported a two-dimensional high-entropy metal phosphorus trichalcogenide Co_0.6_(VMnNiZn)_0.4_PS_3_, which possesses the strengths of tunable adsorption energy and a large specific surface area. The multicomponent high-entropy nanosheets with highly exposed active sites displayed much improved HER performances: an overpotential of 65.9 mV at a current density of 10 mA cm^−2^ and a small Tafel slope of 65.5 mV dec^−1^ (Fig. 5j). DFT calculation and spectroscopy characterization indicated that the moderated S sites on the edge and P sites on the basal plane could offer abundant active sites for intermediate adsorption, and the combined Mn sites also promote the subsequent water dissociation process during the Volmer step (Fig. 5i).

**Fig. 6:**
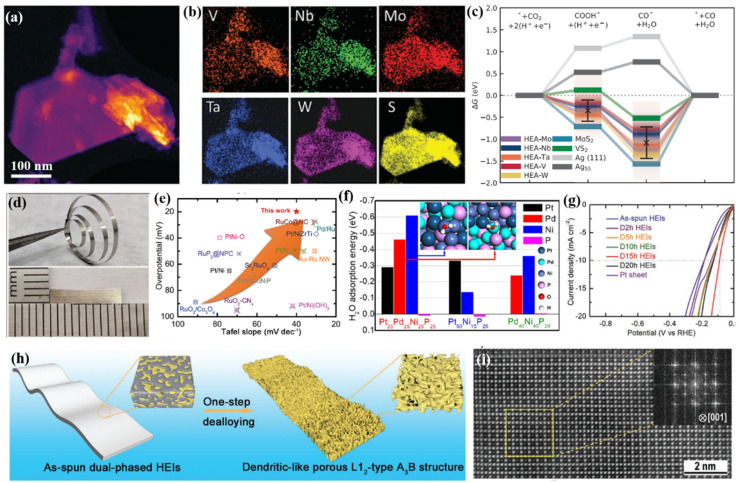
(a) STEM-HAADF image showing a typical high entropy TMDC flake; (b) EDS chemical maps showing V, Nb, Mo, Ta, W, and S distribution for the flake; (c) free energy pathway of CO_2_ reduction at the equilibrium condition potential; (a–c) adapted with permission from ref. 102. Copyright 2021, Wiley-VCH. (d) Photographs of the Pt_25_Pd_25_Ni_25_P_25_ HEMG; (e) comparison of the HER performance with that of recently reported noble-metal-based electrocatalysts in 1.0 M KOH solution; (f) DFT calculated adsorption energies of H_2_O molecules on different exposed elemental sites of Pt_65_Ni_15_P_25_, Pd_40_Ni_40_P_20_, and Pt_25_Pd_25_Ni_25_P_25_; (d–f) adapted with permission from ref. 103. Copyright 2022, American Chemical Society. (g) LSV curves of the prepared samples; (h) the schematic diagram of the dealloying process from a dual-phase structure to a dendritic-like L1_2_ structure; (i) aberration-corrected HAADF-STEM image viewed along the [001] zone axis showing the L1_2_-type A_3_B structure; (g–i) adapted with permission from ref. 105, copyright 2020, Wiley-VCH.

Cavin *et al.*^102^ reported a 2D high-entropy transition metal dichalcogenide with four/five transition metals (Fig. 6a and b). They found that five-component (MoWVNbTa)S_2_ with the highest configurational entropy is highly active and selective for catalyzing CO_2_ conversion to CO, with a high current density of 0.51 A cm^−2^ and a turnover frequency of 58.3 s^−1^ at about −0.8 V *versus* RHE. DFT calculations reveal that the high CO_2_ reduction is due to multi-site catalysis, where the atomic-scale disorder optimizes the rate-limiting step (CO desorption) by facilitating isolated edge sites with weak CO binding (Fig. 6c). Jia *et al.*^103^ found that alloying P with Pt, Pd, and Ni with equal atomic ratios would lead to an amorphous HEA. Pt_25_Pd_25_Ni_25_P_25_ exhibits 5- and 10-fold higher activity for the HER in alkaline solutions when compared with ternary Pt_60_Ni_15_P_25_ and Pd_40_Ni_40_P_20_ amorphous alloys, respectively, and surpasses the performance of Pt/C (Fig. 6d and e). The enhanced activity is due to a synergistic interaction of these four elements, the optimized electronic structure and a large number of unsaturated atomic sites for the HER (Fig. 6f).

Quite recently, Pang and coworkers synthesize a library of Prussian Blue Analogues (PBAs) from binary to multicomponent by a facile co-precipitation method. They found that the high-entropy PBA can not only inhibit the shuttle effect by serving as a polysulfide immobilizer but also act as a catalyst to promote polysulfide conversion, thereby boosting its Li–S battery performance.^104^ Although cation regulation has been widely used so far, the anion regulation certainly provides a new route for the property adjustment for various applications such as batteries, electrolytes, *etc.*, and more novel high-entropy compounds can be expected in future.

### 5.3. Ordered high-entropy intermetallics

Until now, most of the studies on HEM catalysts have been concentrated on one-phase solid-solution structures. However, the disordered crystal structures would limit the underlying atomic configuration of constituent metallic elements and then hinder the display of certain underlying beneficial properties.^79,105,106^ However, multicomponent high-entropy intermetallic nanoparticles are challenging to prepare due to the tendency of phase separation and particle aggregation during annealing. To solve these issues, Hu and coworkers used 5 min Joule heating to promote the phase transition of HEA (*e.g.*, PtPdAuFeCoNiCuSn) nanoparticles into an L10 intermetallic structure.^107^ Wang's group developed an ordered PtRhFeNiCu high-entropy intermetallic, exhibiting outstanding catalytic activity for the ethanol oxidation reaction in comparison with disordered HEAs.^108^ Both DFT calculation and experimental results show that the high-entropy intermetallic has a stronger C–C bond breaking ability than the corresponding disordered HEA and commercial Pt/C, because the ordered structure reduces the energy barrier by changing the adsorption configuration of key intermediates. In another study, Jia *et al.*^105^ revealed that the chemical complexity and remarkable atomic configurations of a quinary FeCoNiAlTi high-entropy intermetallic could provide strong synergistic effects to moderate their electronic structures (Fig. 6h and i). The L1_2_-type ordered structure provides a specific site isolation effect and optimizes H_2_O/H^+^ adsorption/desorption for the HER. As a result, an overpotential of 88.2 mV was obtained at 10 mA cm^−2^ with a Tafel slope of ∼40 mV dec^−1^, which is close to the performance of noble metal-based catalysts (Fig. 6g).

Furukawa and coworkers designed a high-entropy intermetallic (NiFeCu)–(GaGe) by multi-metalizing NiGa intermetallic with a CsCl-type structure.^106^ The high-entropy intermetallic with isolated Ni sites can completely inhibit ethylene overhydrogenation even at complete acetylene conversion and shows 5 times higher activity than other 3d transition metal-based catalysts. DFT calculation suggests that the multicomponent design leads to lower surface energy, which dramatically weakened ethylene adsorption.

## 6. Theoretical calculation/prediction

The tunable compositions of HEMs provide huge possibilities for researchers to develop effective catalysts for various catalytic reactions. However, this also brings about difficulties and challenges to find high-performance HEMs with optimized compositions through experiments alone. Thus, theoretical studies such as density functional theory (DFT) calculation and machine learning may play key roles in the multicomponent catalyst design. Moreover, the DFT calculation is also of considerable significance to help to figure out the electronic interaction of different elements, the synergistic effect and real active sites, which is hard by experiments.

### 6.1. DFT calculation

**Fig. 7:**
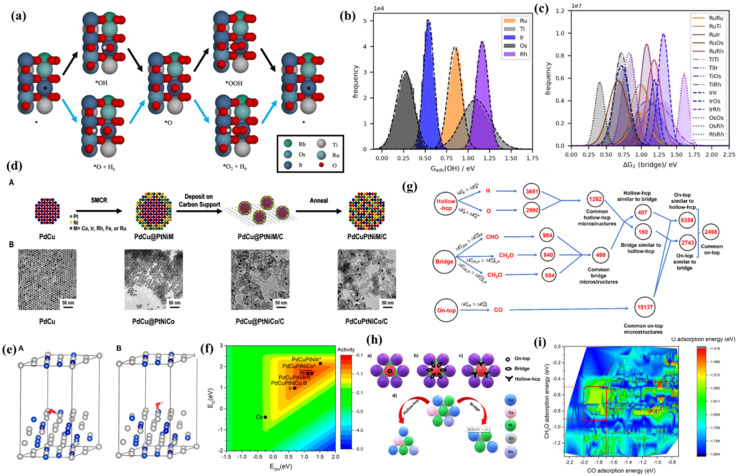
(a) Reaction pathways for the OER: conventional pathway (black arrows) and pathway involving the bridge site (blue arrows); distribution of energies for (b) the adsorption of *OH on all possible cus sites; (c) Δ*G*_2_ on all possible bridge sites as calculated with the fitted model; (a–c) adapted with permission from ref. 121. Copyright 2022, Wiley-VCH; (d(A)) illustration of the three-step process to obtain HEA NPs with their respective TEM images in (B) for the PdCuPtNiCo system; (e) computational models for DFT calculations of (A) O adsorption and (B) OH adsorption on the PdCuPtNiCo surface; (f) estimated oxygen reduction activity as a function of O and OH adsorption energies is plotted along with the specific adsorption energies of PdCuPtNiCo and PdCuPtNiIr (squares) and the pure metals Pt, Ir, and Co (circles); (d–f) adapted with permission from ref. 113, copyright 2022, American Chemical Society; (g) flowchart for finding active and selective catalysts for methanol formation from the CO_2_ reduction reaction through correlation among different adsorption sites; (h) possible (a) on-top, (b) bridge, and (c) hollow-hcp adsorption sites in an on-top surface microstructure and (d) correlation of the on-top microstructure with its corresponding similar hollow-hcp and bridge microstructures; (i) contour plot for the adsorption energy of *CO, *H_2_CO, and *O. The range of O adsorption energy was indicated by a color map, and the region of the selective catalysts was indicated by the red square. (g–i) Adapted with permission from ref. 125, copyright 2022, American Chemical Society.

As is well known, DFT is a powerful weapon to verify the experimental results by calculating the energy of formation of oxygen vacancies, Gibbs free energy difference (Δ*G*) diagrams, density of states (DOS), charge distribution analysis and their behavior under strain.^109–111^ Therefore, the use of reasonable theoretical calculation/prediction could help researchers to develop efficient catalysts more easily from a large composition space. For high-entropy catalysts, the different local atomic environments lead to a distribution of binding energy for the catalytic intermediates, among which specific sites may reach the maximum activity on the basis of the Sabatier principle. Inspired by the high catalytic activity of RuO_2_ and IrO_2_, Svane *et al.*^112^ chose the OER on the (110) surface of a rutile oxide based on Ir, Ru, Ti, Os and Rh as an example, and further put forward a strategy for the theoretical optimization of the composition (Fig. 7a). DFT calculations were performed on a limited number of sites to screen a model that predicts the reaction energy for all possible local atomic environments (Fig. 7b and c). The calculation results indicate that the composition with the highest catalytic activity depends on the assumed reaction path; considering only the conventional pathway, the optimized composition is heavy in Ti. Meanwhile the inclusion of the bridge pathway reveals a mixture of Ru, Ir and possibly a small amount of Rh as the optimum. In another study, Sandra *et al.*^113^ adopted DFT methods to disclose the O and OH adsorption energies of PdCuPtNiCo and PdCuPtNiIr surfaces, which were adopted as computational descriptors for oxygen reduction activity (Fig. 7d). Based on the weighted adsorption energy, it is found that the predicted ORR activity follows the order of Pt ≈ PdCuPtNiCo > PdCuPtNiIr, which is in accordance with experimental results and completely different from the unweighted results (Fig. 7e and f). The finding highlights that the underlying elemental distribution of HEA nanoparticles such as intraparticle heterogeneity which is likely overlooked in many systems can be leveraged toward efficient catalysis.

It should be mentioned that only a limited number of models are studied in most of the DFT studies due to the numerous possibilities. For conventional DFT calculations, it is unpractical to consider every possible atomic configuration to comprehensively study the mechanism. At the same time, the complex atomic configurations and possible lattice distortion in high-entropy catalysts dramatically increase the difficulty of structural optimization and the computation cost. Thus, the development of a novel high-throughput calculation strategy possibly combined with machine learning should be promising for developing new and efficient catalysts.

### 6.2. Machine learning

In recent years, machine learning has attracted much attention because of its incomparable capability to multi-process data and adjustable analytical models to analyze multicomponent materials.^114–117^ Machine learning needs a specific descriptor to assess the corresponding data. Generally, the atomic distribution, electronic structure, and some thermodynamic parameters including the adsorption energy of molecular species and formation energy of optimized structures function as three common descriptors for machine learning.^35^ Besides being widely adopted in single-atom catalysts^118^ and intermetallics,^119^ machine learning is also considered to be very effective for predicting advanced high-entropy catalysts.^120,121^ By using the ORR as a model reaction, Rossmeisl and coworkers present DFT calculated *OH and *O adsorption energies on a random subset of possible binding sites of the IrPdPtRhRu HEA surface.^122^ A simple machine learning algorithm was then used to predict remaining adsorption energies and they found good agreement between the predicted and calculated values. With a full catalog of available adsorption energies, an appropriate expression for predicting catalytic activity was used to optimize the HEA composition. Moreover, the optimization strategy is applicable to any surface reaction where the adsorption energy of an intermediate is the key descriptor, and the model provides an accurate and time-efficient mapping between the adsorption energy and surface structure that is crucial for the success of the optimization. They later used the same strategy (DFT calculation and machine learning) to optimize disordered CoCuGaNiZn and AgAuCuPdPt HEAs for CO_2_ and CO reduction.^123^ Lu *et al.*^124^ leveraged a neural network (NN) and DFT to simultaneously account for the ligand effect (spatial arrangement of different elements) and coordination effect (different crystal facets and defects) for predicting the adsorption energy. Although trained only on DFT data, the machine learning's prediction is in basic agreement with experimentally obtained activity in reported literature, suggesting great potential for HEA catalyst design.

Pathak and coworkers investigated high-throughput screening of high entropy alloy (Cu, Co, Ni, Zn, and Sn) based catalysts through machine learning for CO_2_ hydrogenation to methanol.^125^ They found that the activity and selectivity of these catalysts can be successfully predicted and have screened a series of high entropy-based catalysts (from 36 750 considered catalysts) for CO_2_ hydrogenation to methanol (Fig. 7g–i).

## 7. Catalytic reactions

**Fig. 8:**
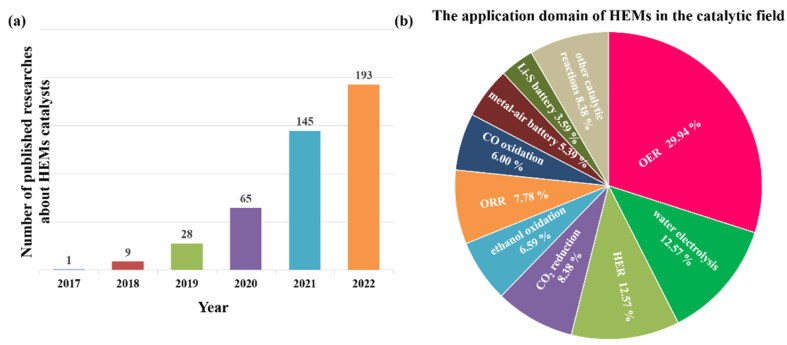
(a) Statistics regarding the number of published articles on HEM catalysts since 2017, and the data are collected from the Web of Science; (b) statistics regarding the application domains of HEM catalysis, and the data used in this pie result from the Web of Science in the last three years.

Since 2018, published research results about high-entropy catalysts increased significantly year by year and in 2022, nearly 200 papers were found in the Web of Science (Fig. 8a). The developed multicomponent high-entropy catalysts have also been applied in many different reactions (Fig. 8b and Table 1). They can be summarized in four categories, which are electrocatalysis (for example, water splitting, fuel cells, metal–air batteries, metal–sulfur batteries, CO_2_ electro-reduction, *etc.*),^17,123,126^ high-temperature gas phase reactions (such as NH_3_ decomposition,^127^ CO oxidation,^128^ CO_2_ hydrogenation,^129^ methane combustion,^130^*etc.*), liquid phase organic reactions,^131,132^ and photocatalytic reactions.^133^ From Fig. 8b, we can see that most of these developed high-entropy catalysts are tried as electrocatalysts for the HER, OER, ORR, metal/ethanol oxidation, CO_2_ reduction, *etc.*, which shows the urgency of the development of fuel cells, water splitting, CO_2_ fixation, metal–air batteries, *etc.* Due to the large amount of literature published, we only show a few references in Table 1. It is noted that designing high-entropy catalysts in photocatalysis may be another important direction although only a few studies are reported. For example, a two-phase oxide with an overall composition of TiHfZrNbTaO_11_ and a d_0_ electronic configuration was prepared, which has an appreciable visible-light absorbance and a 2.9 eV bandgap. The HEO is thus active for photocatalytic water splitting.^133^ In another study, to achieve visible-light-driven photocatalysis, the same group prepared a Ti–Zr–Nb–Ta–W–O system with 10 different heterojunctions by a high-pressure torsion method and oxidation.^134^ Due to its high visible-light absorption, narrow bandgap, suitable band structure, multiple heterojunctions and accordingly facilitated electron–hole separation, the HEO photocatalyst can produce oxygen from water under visible light without a co-catalyst. These results clearly show the potential of high-entropy design to develop new and effective visible-light photocatalysts. It is expected that the further incorporation of noble metal nanoparticles with HEOs can further enhance the photocatalytic efficiency by further enhancing the electron–hole separation.

**Table 1:** Summary of HEMs for electrocatalysis, high-temperature gas phase reactions, liquid phase organic reactions, and photocatalytic reactions

Material	Structure	Synthetic method	Catalytic reaction	Ref.
CoFeNiMnMoPi	Rock salt	Rapid heat treatment	OER	17
FeCoNiPB/(FeCoNi)_3_O_4−*x*_	Amorphous	Melt spinning method	OER	13
FeCoNiMo	FCC	Solid phase reaction	OER	135
(MoWVNbTa)C	*Fm*3̄*m*	Electrical discharge process	HER	101
FeCoNiAlTi	L1_2_-type HEI	Dealloying method	HER	105
(Cu,Ni,Co,Fe,Mn)_3_O_4−*x*_\C	Spinel	Carbothermal shock	ORR	75
AlAgAuCoCuFeIrMoNiPdPtRhRuTi	FCC	Dealloying method	HER/OER	42
CoFeNiCrV (oxy)hydroxide	Rhombohedral hydrotalcite	Anodic oxidation approach	Water splitting	31
AuAgPtPdCu	FCC	Melting and cryogrinding	CO_2_ electro-reduction	6
(CoCrFeMnNi)_3_O_4_	Spinel	Soft chemical approach	Methanol electrooxidation	8
AlPdNiCuMo	FCC	Dealloying method	Ethanol electrooxidation	30
MgZnNiCuCo oxides	Rock salt	Ball milling	Li–sulfur battery	126
AlNiCoRuMoCrFeTi oxides	Spinel	Dealloying	Zn–air batteries	98
PtPdAuRu/CNF	FCC	Carbothermal shock	Li–O_2_ battery	136
CoMoFeNiCu	FCC	Carbothermal shock	NH_3_ decomposition	127
(CeLaPrSmY)O_2−*y*_	Fluorite	Sol–gel method	CO oxidation	128
Co_3_MnNiCuZnO_*x*_	Spinel	Mechanochemical redox	CO_2_ hydrogenation	129
(ZrCeHfTiLaYGdCaMgMn)O_2−*x*_	Fluorite	Carbothermal shock	Methane combustion	130
ZnCoCdNiCu-ZIF	ZIF	Ball milling	CO_2_ cycloaddition	16
Ce_0.2_Zr_0.2_La_0.2_Pr_0.2_Gd_0.2_O_2_	Fluorite	Sol–gel method	1,2-Diketones from aldehyde	131
Co_0.2_Ni_0.2_Cu_0.2_Mg_0.2_Zn_0.2_O	FCC	Anchoring and merging process	Aerobic oxidation	132
TiHfZrNbTaO_11_	Monoclinic/orthorhombic	Mechanical alloying/oxidation	Water photolysis	133

## 8. Summary and perspectives

In this review, we mainly discussed the design strategies of HEMs and summarized the recent research progress of HEMs as catalysts/electrocatalysts/photocatalysts. However, the relationships between the structural effects and catalytic properties of HEMs are still unambiguous, putting obstacles in the way of purposely regulating the intrinsic surface electronic states and catalytic activities. It is also worth mentioning that so far we cannot mix any five or more components in one nanostructure without phase separation, especially for these enthalpy-dominated systems. Some specific perspectives and outlooks are shown below:

(1) Although high entropy is usually used for the description of these multicomponent catalysts, it should be point out that the entropy value is usually not related to the catalytic activity. The high entropy does contribute to the enhanced stability of the system considering that Δ*G* = Δ*H* − *T*Δ*S*.

(2) Normally, for a high-entropy or multicomponent catalyst to become a necessity, the high-entropy or multicomponent catalyst should be better than all these fewer component catalysts. For example, if a quinary HEA has the best catalytic activity, in theory, its performance should be better than that of all the unary, binary, ternary, and quaternary samples and the quinary sample with other element combinations and more component samples (more than 5 components). However, due to the numberless combinations, a thorough comparison cannot be achieved in most cases. Thus, in all these published studies, only limited comparisons are carried out. In other words, the reported “best” catalyst may not be the best one. Considering this, a high throughput fabrication strategy is highly needed to prepare more samples for catalyst screening.

(3) Based on the literature and our understanding, normally, the following experimental designing strategies could be used. (1) It is known that Pt-group noble metals are very active for certain reactions such as the HER, MOR, *etc.* Thus, mixing at least five Pt-group noble metals in one nanostructure may result in enhanced catalytic activity than any of these pure metals. (2) Usually, noble metals are the active sites for many reactions. We then mixed one or two noble metals with four or three non-noble elements to see if the high-entropy mixing of these inert non-noble elements can enhance the activity of the noble metal active sites. (3) This strategy (*i.e.*, based on the well-known unary, binary or ternary systems, and then mixing more elements to reach five or more component systems) is also extended to non-noble metal active sites. For example, Ni and Co are well-known active sites for the OER. We then mixed Ni and Co with other elements (such as Fe, Mo, *etc.*) to further enhance the OER activities of Ni/Co sites. It should be mentioned that justifying the necessity of using a high-entropy catalyst is still not an easy task considering the quite limited comparison samples. On the other hand, mixing five or more inert elements to generate an active catalyst is an interesting research direction although very challenging.

(4) If noble metals are involved in a high-entropy sample and considering that the noble metal is usually the active site for many reactions, the high-entropy catalyst design can greatly reduce the noble metal content. Thus, if a high-entropy sample (such as Pt or Ru containing HEMs) achieves a comparable activity with Pt/C or RuO_2_, we would consider the high-entropy design a success. As long as the high activity is retained, the more non-noble element incorporated, the more cost effective the designed catalyst.

(5) At present, the choice of element combination is basically based on experience and trials & errors. Thus, developing a novel strategy such as high throughout DFT calculation and machine learning to determine the element combination for a specific chemical reaction is very urgent.

(6) At present, most studies focus on the fabrication of single phase high-entropy nanomaterials for catalysis. However, for a specific reaction, the phase or element separation may result in a better activity considering the rich interface and possible stepwise reaction on different sites of the heterostructure.

(7) The real active sites of high-entropy catalysts for many reactions are still misty, especially for more complicated high-entropy systems such as HEOs/noble metal composites. Thus, more *in situ* characterization techniques such as X-ray absorption spectroscopy are needed to study the electronic state changes of each surface element.

(8) Based on these understandings, the following directions may be appealing for the future of multicomponent high-entropy catalysts. (1) Developing an advanced synthesis strategy for the preparation of single-crystal HEMs (single-crystal HEMs are extremely hard to achieve due to the extensive existence of crystalline lattice strains, lattice distortions, and numerous unsaturated defects) and high-entropy materials with precisely controlled nanostructures (such as, with selectively exposed highly active facets or selective distribution of specific elements on a near-surface of HE crystals). (2) Due to the endless combination of elements and constituents, it is necessary to develop high throughout synthesis or DFT calculation strategies to screen the most efficient catalysts with synergistic effects between elements. (3) It is challenging to prepare single atom catalysts with ultrahigh loading *via* a simple and convenient method. However, the rich and tunable chemical coordination environment in HEMs makes it possible to incorporate a large amount of single atom catalysts, which would maximize the utilization of metals and facilitate atomic interaction studies. (4) *In situ* monitoring of the surface atomic/electronic structural changes and elemental distribution change of high-entropy catalysts by using advanced characterization technique such as *in situ* environmental high-angle annular dark-field (HAADF) scanning transmission electron microscopy (STEM), X-ray absorption near-edge structure (XANES) spectroscopy, *etc.* (5) Incorporating more elements such as more than 12 or 14 elements into one nanosystem considering the fact that most atoms are in single atom states and surrounded by completely different atoms.

In summary, developing high-entropy or multicomponent catalysts is still in an early stage. The reported so-called best high-entropy (multicomponent) samples may still need further optimization. The surface states may be completely different from the bulk composites and the surface may be severely oxidized if many active elements are incorporated. Thus, real-time surface state characterization is very important. One should also keep in mind that for a multicomponent catalyst, the atomic ratios of each element should also be examined/optimized. An equal atomic ratio does not always lead to the highest activity although the highest entropy value is achieved. In any case, it is expected that by high-entropy design (*i.e.*, incorporating more elements into one system) and exploring the synergistic effect, we can further enhance the catalytic performance (both activity and stability) of a catalyst. Or we can significantly reduce the noble metal content (*i.e.*, lowering the cost) without sacrificing much activity if the noble metal is indispensable. It is also hoped that multicomponent catalysts can greatly enhance the reaction selectivity by changing the conventional reaction path, which is very important for organic synthesis.

## Author contributions

H. Xu and Z. Jin contributed equally to this work. The manuscript was written by H. Xu and H.-J. Qiu. H.-J. Qiu revised and supervised the manuscript. All authors discussed this manuscript.

## Conflicts of interest

The authors declare that they have no known competing financial interests or personal relationships that could have appeared to influence the work reported in this paper.

## Supplementary Material
